# Autoencoder Networks Decipher the Association between Lung Cancer and Alzheimer's Disease

**DOI:** 10.1155/2022/2009545

**Published:** 2022-12-05

**Authors:** Jialin Li, Xinliang Gao, Mingbo Tang, Chi Wang, Wei Liu, Suyan Tian

**Affiliations:** ^1^Department of Thoracic Surgery, The First Hospital of Jilin University, 1 Xinmin Street, Changchun, Jilin 130021, China; ^2^Department of Internal Medicine, College of Medicine, Markey Cancer Center, University of Kentucky, 800 Rose Street, Lexington, KY 40536, USA; ^3^Division of Clinical Research, The First Hospital of Jilin University, 1 Xinmin Street, Changchun, Jilin 130021, China

## Abstract

Lung cancer is the most common malignancy and is responsible for the largest cancer-related mortality worldwide. Alzheimer's disease is a degenerative neurological disease that burdens healthcare worldwide. While the two diseases are distinct, several transcriptomic studies have demonstrated they are linked. However, no concordant conclusion on how they are associated has been drawn. Since these studies utilized conventional bioinformatics methods, such as the differentially expressed gene (DEG) analysis, it is naturally expected that the proportion of DEGs having either the same or inverse directions in lung cancer and Alzheimer's disease is substantial. This raises the inconsistency. Therefore, a novel bioinformatics method capable of determining the direction of association is desirable. In this study, the moderated *t*-tests were first used to identify DEGs that are shared by the two diseases. For the shared DEGs, separate autoencoder (AE) networks were trained to extract a one-dimensional representation (pseudogene) for each disease. Based on these pseudogenes, the association direction between lung cancer and Alzheimer's disease was inferred. AE networks based on 266 shared DEGs revealed a comorbidity relationship between Alzheimer's disease and lung cancer. Specifically, Spearman's correlation coefficient between the predicted values using the two AE networks for the Alzheimer's disease test set was 0.825 and for the lung cancer test set was 0.316. Novel bioinformatics methods such as an AE network may help decipher how distinct diseases are associated by providing the refined representations of dysregulated genes.

## 1. Introduction

Lung cancer is the most common malignancy in humans and causes the largest cancer-related mortality worldwide [1]. Of the two major subtypes, non-small cell lung cancer and small cell lung cancer, the former accounts for almost 80% of cases and can be further divided into two subtypes, lung adenocarcinoma and lung squamous cell carcinoma [2]. Alzheimer's disease is a degenerative neurological disease. It is the most common type of dementia, accounting for approximately 60–80% of patients with dementia [3]. While approved therapeutics show only mild effects on halting the disease's progression [4], advanced methods such as different frequency electromagnetic fields (EMF), which have been suggested by a previous review [5] to be beneficial to Alzheimer's disease, are far away from the clinical utilization due to discrepancies and shortages of well-designed experimental validation.

Although lung cancer and Alzheimer's disease are distinctly different, studies have suggested that they are linked. For example, several epidemiologic studies [6–8] have demonstrated a risk reduction for Alzheimer's disease after a cancer diagnosis (including lung cancer) [9–11]. Moreover, transcriptomic studies [12–14] have indicated that the genes upregulated in Alzheimer's disease and downregulated in cancer, as well as the genes downregulated in Alzheimer's disease and upregulated in cancer were significantly overlapped. In addition, expression deregulation in opposite directions was observed at the level of pathways in Alzheimer's disease and cancer [15]. These observations give some support to the idea of an inverse correlation between Alzheimer's disease and cancer.

While the opposing pathological processes (for example, uncontrolled cell proliferation in cancer versus neuronal cell death in Alzheimer's disease) [16] provide a partial explanation for this inverse relationship, one may argue that the competing risk of death in patients with cancer may drive the estimation of the association coefficient towards a negative direction [17]. Conversely, a few observational studies suggested no association [18], or a very weak negative association [19], or a positive association between these two diseases [20–22].

Moreover, all relevant transcriptomic studies have explored the association between the two diseases by using conventional bioinformatics methods, such as differentially expressed genes (DEGs), pathway enrichment analysis [23], and the weighted gene coexpression network analysis [24]. Such analyses have major drawbacks. For instance, for DEG analysis, it is naturally expected that all DEGs have identical/inverse regulation directions. This makes the determination of association direction very difficult. All recent studies examining the relationship between Alzheimer's disease and lung cancer are summarized in Table 1, from which it is obvious that no concordant conclusions have been obtained, thereby leaving the question unsolved.

Deep learning methods [25] hold promising capacity for dealing with high-dimensional data, and are widely applied to analyze data of certain complex diseases. For example, Ramana et al. [26] proposed a novel model combining deep learning and the capsule network, which has been shown to possess better discriminative ability when applied to lung cancer CT image data. Moreover, Lee and Lee [27] utilized fully connected neural networks (FNN) to predict the risk of developing Alzheimer's disease based on gene expression profiles. Even more, a deep learning method can be deployed in a wearable device or a smartphone (the underlying framework is built upon a deep learning method) to monitor an individual's health status or a patient's symptoms, e.g., [28] for an early detection and intervention of certain diseases.

Autoencoder (AE), a deep learning method, has been widely used in the realm of omics data analysis [29], especially transcriptomic data. It consists of two parts: an encoder and a decoder. The encoder compresses the data into a low-dimensional vector, which is regarded as a hidden representation of the data. The low-dimensional vector is then uncompressed by the decoder to obtain reconstructed data in a way that mimics the original data as precisely as possible (to represent the signals in the original data) but they have a refined dimension (thus, noise can be discarded). Considering AE can accomplish the task of generating a lower-dimensional representation for an individual's gene expression profile without any difficulties and it is simpler compared to many other deep learning methods, we used the AE method to embed the DEGs into a one-dimensional space while preserving their gene-to-gene interplay to further explore how lung cancer and Alzheimer's disease are associated. Since the output of the encoder in this study is a single vector, a unified answer to the question of how these two diseases are related is possible. To the best of our knowledge, no studies have integrated both deep learning and omics data mining to specifically explore the relationship between the two diseases.

## 2. Materials and Methods

### 2.1. Experimental Data

Raw data of the Alzheimer's disease cohort used in the study were downloaded from the Gene Expression Omnibus (GEO: https://www.ncbi.nlm.nih.gov/geo/) repository (under accession numbers GSE4757, GSE 48350, and GSE5281) [30–32]. Microarray experiments for the lung cancer cohort were those studies stored under accession numbers GSE18842, GSE102287, GSE19804, GSE19188, GSE103888, and GSE118370 [33–38].  Table 2 summarizes the demographic characteristics of the microarray data considered in this study.

Since the output of the encoder is a vector, the depth is required to be relatively high to extract the largest amount of useful information from the original data while allowing for good generality. Correspondingly, we combined several microarray datasets into an integrated dataset in both the Alzheimer's disease and lung cancer cohorts to enlarge the sample size. The inclusion criteria were the chips profiled on the Affymetrix HG-U133 Plus 2.0 platform; the sample size was larger than 10; and the ratio of cases and controls approximately ranged from 0.5 to 2.

### 2.2. Preprocessing Procedure

The fRMA algorithm was utilized to preprocess raw data. Compared to other pre-process methods (e.g., GCRMA and RMA), fRMA can effectively control or eliminate batch effects and provide summary expression values for a single array. Of note, the ability to control or eliminate the batch effect is especially relevant to the current study since multiple studies were involved in both the lung cancer and Alzheimer's disease cohorts. When multiple probe sets were matched to the same gene, the probe set with the largest absolute log-fold change between the diseased group and the control group was kept. Finally, the Combat algorithm was implemented to eliminate the possible remaining batch effects.

### 2.3. Identification of DEGs

Moderated *t*-tests (executed by the R limma package) were conducted to identify the differentially expressed genes, and the *p* values were adjusted for multiple testing using the Benjamini–Hochberg (BH) procedure to obtain false discovery rate values. The cutoff values for false discovery rate and log fold change were 0.05 and 0.5, respectively.

### 2.4. AE Models Used to Extract a One-Dimensional Summary Score

The deep learning process was implemented on the overlapped DEGs to extract a one-dimensional representation (that is, a pseudogene to represent all identified overlapped DEGs). Based on the pseudogene, the association direction between lung cancer and Alzheimer's disease were inferred. The whole dataset (including all respective cases in the integrated datasets) was randomly divided into a training set and a validation set with a ratio of 3 : 2, in which the ratio of cases to controls are roughly equal.

The encoder of the AE model comprised a dense network connecting the input layer to a hidden layer with 128 nodes, a second layer with 64 nodes, and a third hidden layer with 10 nodes. The activation function used in the encoder network was Rectified Linear Unit (ReLU). Then the third hidden layer was connected to a bottleneck layer with one node. The dropout rate was set at 0.2.

The decoder of the AE model comprised inversely ordered layers beginning with a fully connected layer that connects the bottleneck layer to a 10 node-hidden layer, a second densely connected layer with 64 nodes, a third layer with 128 nodes, and the output layer which decodes back the dimension of original data without any activation function (linear transformation). Again, the dropout rate was set at 0.2. The AE model was optimized using a stochastic gradient descent method (the Adam method), and the learning rate was set at 0.0005.

The aforementioned values of hyper-parameters were calculated via a grid search, and the optimal values were configured based on those minimizing mean squared error (MSE). Specifically, the learning rate was selected from 0.0001, 0.0005, 0.001, and 0.002, and the depth of AE network (including the output layer) was tried from 6, 8, and 10. Three values, 64, 96 and 128, were tried for the node number of the first hidden layer; 32, 48, and 64 for the second hidden layer; 10, 12, and 16 for the third hidden layer. The dropout rate was selected from 0.1, 0.2, and 0.3. Default values were retained for the remaining hyperparameters. The training processes were set to stop, and related model parameters were confirmed if the MSE metrics in the validation sets showed marginal changes/decrements.

**Figure 1:**
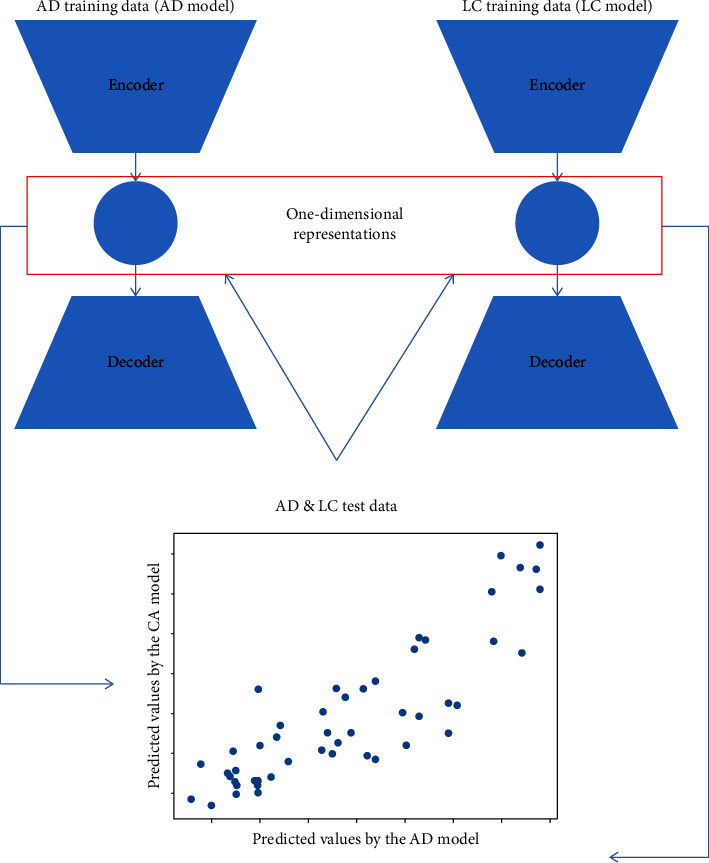
Flowchart illustrating how the proposed procedure (based on autoencoder networks) provides a unified answer to the association direction between the lung cancer and Alzheimer's disease. LC: lung cancer, AD: Alzheimer's disease, AE: autoencoder.

Next, using the AE network for lung cancer, the representation scores of Alzheimer's disease patients and lung cancer patients (using the respective test sets) were calculated. Similarly, using the AE model for Alzheimer's disease, the representation scores of lung cancer patients and Alzheimer's disease patients (again, using the data from test sets) were calculated. Of note, the predicted values for lung cancer/Alzheimer's disease patients calculated using the weights learned from the deep learning model for the opposing diseases are counterfactual. Scatterplots of the predicted values based on the Alzheimer's disease AE model versus the predicted values based on the lung cancer AE model were diagrammed to examine how Alzheimer's disease and lung cancer are related. The flowchart of the proposed procedure is presented in Figure 1. Lastly, the Python codes of AE modeling have been restored in the GitHub repository (https://github.com/windytian/AE_geneexpression).

### 2.5. Pathway Enrichment Analysis

String software (https://www.string-db.org) was used to obtain the gene-to-gene interaction networks for the overlapped DEGs between Alzheimer's disease and lung cancer using a cutoff of confidence scores at 0.7 [39]. The resulting files that recorded these networks were then uploaded into the Cytoscape software for visualization and subsequent hub-gene searching. The Cytoscape plugin, CytoHubba [40], was utilized to identify the hub genes that may stand in the essential positions in the resulting networks. Here, the top 50 genes ranked by their connectivity degree were regarded as the hub genes. The pathway enrichment analyses to obtain the KEGG pathways [41] and GO terms [42] enriched by the identified DEGs of lung cancer and Alzheimer's disease were carried out using the R clusterProfiler package. Except for the cutoff value of FDR loosened to 0.2, the default values of other parameters in the clusterProfiler package were used.

The GeneCards (https://www.genecards.org) [43] knowledge base and PubMed were searched to investigate the biological relevance of identified overlapped DEGs with unique gene symbols.

## 3. Software

All experiments were executed using the Python 3.6 programming language and the R 4.1.1 version. The fRMA algorithm was utilized to preprocess the raw data [44]; the combat algorithm (implemented by the R SVA package) [45] was used to adjust for batch effect; limma was used to fit moderated *t*-tests; clusterProfiler was used to carry out pathway enrichment analysis; and ggplot2 was used to draw bulb plots.

In addition, the Python Keras library in the framework of TensorFlow was used to implement AE networks.

## 4. Results

### 4.1. Identification of Respective DEGs and Overlapped DEGs

The integrated lung cancer cohort included 464 subjects, comprising 251 lung cancer patients and 213 normal controls/tissues. Using the whole integrated lung cancer dataset, the moderated *t*-tests were carried out to identify the DEGs between the diseased group and the control group. In this comparison, 1,935 genes were identified as downregulated and 1,353 as upregulated DEGs.

In the Alzheimer's disease cohort, 177 patients and 257 controls were included. Likewise, moderated *t*-tests were used to identify DEGs. In the comparison between the Alzheimer's disease group and the control group, 508 DEGs were upregulated and 263 were downregulated.

The number of overlapped genes between these two sets of DEGs is 266, of which 21 genes were co-upregulated in the two cohorts and 75 genes were co-downregulated. The other 170 genes were inversely expressed. Notably, both concordantly regulated genes (36%) and inconcordantly regulated genes (64%) account for substantial proportions of the overlapped DEGs, which made the inference on the association direction between Alzheimer's disease and lung cancer inconsistent.

### 4.2. Pathways Enriched by the Overlapped DEGs

**Figure 2:**
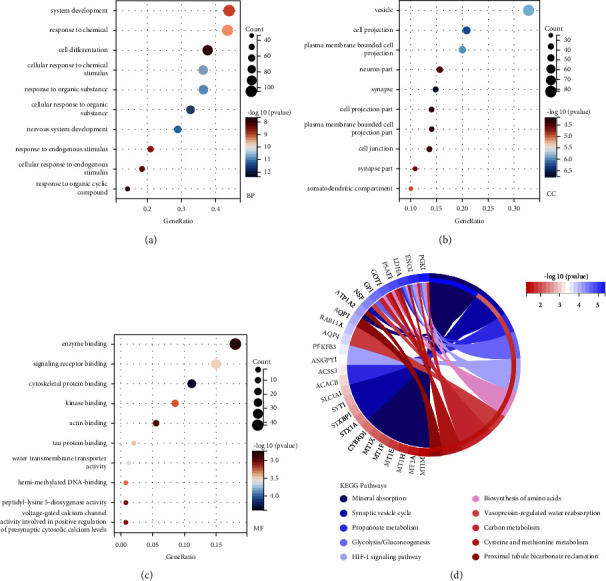
Pathway enrichment analysis of overlapped DEGs of lung cancer and Alzheimer's disease cohorts. (a) The gene ontology biological process analysis of overlapped DEGs. (b) The gene ontology cellular component of overlapped DEGs. (c) The gene ontology molecular function analysis of overlapped DEGs. (d) The KEGG analysis of overlapped DEGs.

Between the Alzheimer's disease and lung cancer cohorts, 266 overlapped DEGs were identified. GO functional analysis showed the overlapped genes were mainly enriched in system development, cell projection, and enzyme binding (Figures 2(a)–2(c)). Enrichment in mineral absorption, the HIF-1 signaling pathway, and carbon metabolism of the KEGG pathway analysis was shown in Figure 2(d). Additionally, the gene-to-gene interaction network that summarizes how interplay of the 266 overlapped DEGs was constructed using String software. The resulting network comprised two large subnetworks (involving more than 10 genes) and several small subnetworks (with at most several nodes). FGF2, SNCA, and LDHA are located in the centers of these two large subnetworks and were identified as hub genes.

### 4.3. Deep Learning Analysis

**Figure 3:**
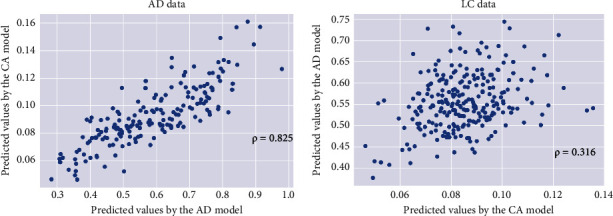
Scatterplots indicating that Alzheimer's disease and lung cancer may be positively correlated. (a) The predicted summary/representation values by the Alzheimer's disease autoencoder model versus those by the lung cancer autoencoder model for patients with Alzheimer's disease based on 266 DEGs. (b) The predicted summary/representation values by the Alzheimer's disease autoencoder model versus those by the lung cancer autoencoder model for patients with lung cancer based on 266 DEGs. LC: lung cancer, AD: Alzheimer's disease.

To obtain a unified estimate on the association between lung cancer and Alzheimer's disease, the AE networks (as described in the methods section) were constructed based upon the expression profiles of these overlapped DEGs, and the outputs of the encoder network (one-dimensional values in the bottleneck layer) were used to evaluate the correlation between the two diseases. The Spearman's correlation coefficient between the predicted values using the two AE networks was 0.825 (*p* < 0.001) for the Alzheimer's disease test set and 0.316 (*p* < 0.001) for the lung cancer test set. The deep learning analysis indicted a positive correlation between Alzheimer's disease and lung cancer (Figure 3).

**Figure 4:**
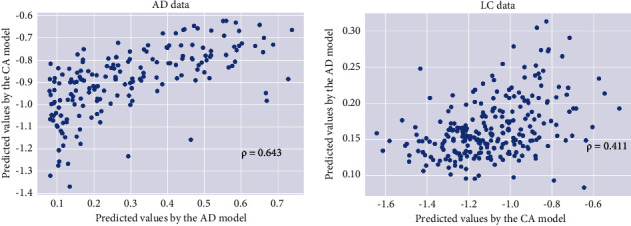
To further verify the positive correlation between Alzheimer's disease and lung cancer using the genes involved in the enriched KEGG pathways. (a) The predicted summary/representation values by the AD autoencoder model versus those by the LC autoencoder model for patients with Alzheimer's disease based on the genes involved in the enriched pathways of respective AD and LC cohorts. (b) The predicted summary/representation values by the LC autoencoder model versus those by the AD autoencoder model for patients with lung cancer based on the genes involved in the enriched pathways of respective LC and AD cohorts. LC: lung cancer, AD: Alzheimer's disease.

Next, we used the respective genes involved in the enriched KEGG pathways for the Alzheimer's disease and lung cancer cohorts to train AE models again and thus to investigate the association between the two diseases more deeply. The Spearman's correlation coefficient between the predicted values using the two AE networks for the test set of Alzheimer's disease was estimated as 0.643 (*p* < 0.001), and for the lung cancer test set was 0.411 (*p* < 0.001) (Figure 4). Again, a positive correlation between the Alzheimer's disease and lung cancer was implied. Notably, the correlations between the predicted values by two AE models were significantly smaller for the lung cancer data. This may be because lung cancer is a very heterogeneous disease. On average, it took 22 seconds for a single run of AE modeling on a Lenovo laptop with an AMD Ryzen 7 4800 U processor and 16 GB RAM.

## 5. Discussion

Insights on the connections between distinct diseases offer new opportunities to uncover their etiology and facilitate drug repurposing. To the best of our knowledge, however, few studies have investigated the association between different diseases from the perspective of molecular biology. This may be because the common relevant genes of two diseases involve genes with identical regulation directions and genes with opposite regulation directions. In this study, we used a deep learning method to generate a one-dimensional representation (a pseudogene) of identified DEGs and then sought to determine the association direction between two diseases based on these pseudogenes.

This study, one of the first attempts to integrate both deep learning and omics data mining to explore how the two diseases are associated, revealed a comorbidity relationship between Alzheimer's disease and lung cancer. Our analysis results differ from the results of most epidemiological studies such as [7, 8]. Potential selection bias may partially explain this inconsistency. Specifically, the prevalence of Alzheimer's disease is directly related to age, and is significantly higher among people >65 years old [46]. However, because 57% of lung cancer cases are diagnosed at late stages with metastasis, of which the 5-year survival rate is only 5% [1], the 5-year survival rate of lung cancer is as low as approximately 19%. Consequently, the relevant epidemiological studies that investigate the relationship between Alzheimer's disease and lung cancer may be subject to survivor bias (selection bias), which deviates the association estimate towards a negative value. On the other hand, the development of lung cancer among Alzheimer's disease patients may be neglected because of these patients' incapability to express their health conditions and corresponding symptoms.

### 5.1. Biological Relevance

As indicated by the GeneCards database, 148 of the overlapped DEGs are directly related to lung cancer and 116 are indirectly related to lung cancer. If the disease of interest is broadened to include all types of cancer, the number of directly relevant genes increases to 258. In contrast, for Alzheimer's disease the number of directly relevant genes is 146 and the number of indirectly relevant genes is 118. Therefore, the identified DEGs have good biological implications, may take part in the cancer and neuron cell differentiation, which may most likely represent the important features of lung cancer and Alzheimer's. Of the 22 upregulated DGEs, DDIT4 [47, 48], FAT1 [49, 50], HSPB1 [51, 52], ZIC2, and SPP1, most among these genes defined as hub genes, are broadly expressed in the nervous system and tumor tissues, and may examine the functional contribution during neuronal differentiation, neuronal death, as well as correlate with malignant biological behaviors of lung cancer. Notably, ZIC2, which represses primary neurogenesis and modulates primary neurogenesis apoptosis in the neural plate [53], is typically overexpressed in Alzheimer's disease and lung cancer. In lung adenocarcinoma, ZIC2 upregulates OCT4 expression to promote cancer stem cell traits, leading to tumorigenesis and a poor prognosis [54].

SPP1 may act as a putative tissue repair gene and work together with other putative tissue repair genes and specialized subgroups overexpressing MHC type II to compose the activated response microglia [55], which is regarded as the converging point for aging, sex, and genetic Alzheimer's disease risk factors. SPP1 is also considered as a marker for highly malignant lung cancer [56]. It is significantly overexpressed in tumor tissues, and may promote the proliferation, migration and invasion of lung cancer cells.

Similarly, some concordant genes were also discovered among the 75 downregulated DEGs, and these genes play critical roles on inhibiting tumor growth and metastasis, as well as improvement of cognitive decline in Alzheimer's disease. For instance, the expression level of BDNF, defined as a hub gene in this study, is decreased in Alzheimer's disease by lowering the phosphorylated cyclic adenosine monophosphate (cAMP) response element binding (CREB) protein, which may lead to synaptic dysfunction and cognitive impairments [57]. In non-small cell lung cancer, miR-496 targeted BDNF-mediated PI3K/Akt signaling pathway suppresses tumorigenesis [58]. CNR1, DHCR24, DPP6, and MEF2C were strongly correlated with disturbances in executive functioning, episodic memory, and visuospatial functioning [59–62], which deregulate expression in multiple human cancers contributing to the antioxidant and repairing activity [63, 64].

At the pathway level, the enriched KEGG pathways and GO terms generated by the 266 overlapped genes were related to nervous system development, cell differentiation, and response to endogenous stimulus, cell junction, and kinase binding. Of them, hypoxia-inducible transcription factor-1 (HIF-1) signaling pathway and hemi-methylated DNA-binding are implicated in the comorbidity observed in lung cancer and Alzheimer's disease. Specifically, HIF-1 controls the response to hypoxia at the molecular level. Hypoxia regulates the activation of HIF by protein stability, phosphorylation [65], nuclear translocation and activity, and consequently mediates Alzheimer's disease progression [66]. Meanwhile, HIF-1-mediated signaling has been implicated in both cell survival and cell death pathways. The HIF-1 pathway participates in promoting metabolic reprogramming by transactivating multiple hypoxia-responsive genes related to glycolytic metabolism [67]. Its activation is a notable characteristic of tumor and contributes to the aggressive biological behavior of lung cancer cells, and relates to a poor clinical outcome [68].

Several previous studies [20–22] shown in Table 1 vouch for a positive association between Alzheimer's disease and lung cancer, which is in harmony with our finding. In this study, the AE networks were constructed to generate respective one-dimensional representations of gene expression profiles for Alzheimer's disease and lung cancer. This computer-aided exploration based on a deep learning consumed less time and generated a unified answer to how the two diseases are related. To our knowledge, this is the first study that integrates a representative learning method and gene expression profiles to specifically explore the association between the two diseases, which is of empirical significance in terms of mining high-dimensional big data and revealing physiopathological mechanisms of complex diseases and their potential association.

Certainly, this study has its own limitations. First, the sample sizes of both the Alzheimer's disease and lung cancer cohorts were not large, which may limit the generalization of the findings from this study. This may be considered as the biggest challenge we face. Second, while AE can accomplish the task of generating a lower-dimensional representation for an individual's gene expression profile without any difficulties, it is an unsupervised learning method that is not excellent at distinguishing among different groups. Finally, the development and progression of certain complex diseases resulted from interaction effects of genetic and environmental factors. The current AE modeling does not include any potential environmental factors.

## 6. Conclusions

The joint analysis of gene expression profiles from Alzheimer's disease and lung cancer based on a deep learning method allowed us to determine the direction of association between the two diseases and then to propose research hypotheses for experimental justification and validation. It is anticipated that deep learning methods should be powerful tools in the relevant research areas. For example, in the future we may generate “digital twins” of Alzheimer's disease/lung cancer patients and computationally mimic the treatment effects of drugs combating these diseases on synthesized patients with the aid of deep learning methods. Additionally, the proposed AE model may be used as the framework to generate an app, thus a clinician can determine the risk of concurrence of the two diseases for an early prevention and intervention based on a patient's gene expression profiles.

As far as the methodology aspect is considered, we will try to collect more gene expression data and include manifold data from other platforms (e.g., RNA-Seq data, proteomics data, and metabolomics data) to investigate the association between the two diseases more deeply. Moreover, we will definitely consider other deep learning methods capable of the dual tasks (enabled to both generate low-dimensional representations and have a good capacity of learning labels, such as a deep graph network equipped with multiple-headed attention mechanisms) and use such a method as the framework to assess the association between Alzheimer's disease and lung cancer again. Lastly, we also plan to include disease-related environmental factors in the final model. More importantly, fundamental and clinical experiments are highly desirable to explore the potential intrinsic mechanisms that can validate and explain the positive link between the two diseases. These studies would pave the way towards drug repurposing and drug combination strategies for the two diseases, and thus, lead to the successful defeat of Alzheimer's disease and lung cancer.

To conclude, deep learning methods such as an AE network may help decipher how the distinct diseases are associated and facilitate drug repurposing. Such an application will save resources and accelerate the clinical implementation of the existing drugs for repurposing.

## Figures and Tables

**Table 1:** Recent studies that investigated the association between the lung cancer and Alzheimer's disease.

Studies	Data sources	Study designs	Statistical methods	Findings
Realmuto et al. [19]	Patients in Palermo, Italy	Case-control	Conditional logistic regression analysis, Mantel–Haenszel analysis	Inverse relationship
Ou et al. [7]	National Health Insurance Research Database in Taiwan	Retrospective	Cox proportional hazards regression, likelihood ratio test	Inverse relationship
Musicco et al. [8]	Health Authority Registry in in Northern Italy	Retrospective	Student's *t*-test	Inverse relationship
Freedman et al. [18]	Medicare and National Cancer Institute's SEER	Case-control study	Logistic regression, Cox proportional hazards regression	No relationship
Feng et al. [21]	International Genomics of Alzheimer's project (IGAP) and Genetic Associations and Mechanisms in Oncology	Genome-wide association study (GWAS)	LDSC package for cross-trait LD score regression	Positive correlation
Sánchez-Valle et al. [23]	GEO and TCGA databases	Meta-analysis based on gene expression data	MetaDE package for the differential expression analysis, gene set enrichment analysis	Inverse relationship
Lee et al. [10]	Korean National Health Insurance Service	Population-based longitudinal study	Cox proportional hazard regression	Inverse relationship
Greco et al. [13]	GEO and TCGA databases	Gene expression data	Stabilized independent component analysis, Wilcoxon test	Inverse relationship
Seddighi et al. [14]	IGAP	Mendelian randomization	Two-sample Mendelian randomization	Inverse relationship
Sherzai et al. [9]	National inpatient sample (of U.S)	Cross-sectional	Logistic regression	Inverse relationship
Forés-Martos et al. [24]	GEO and TCGA databases	GWAS	Cross-trait LD score regression	Inverse relationship
Karanth et al. [6]	University of Kentucky Alzheimer's Disease research center and Kentucky cancer Registry (mainly people in Kentucky, US)	Retrospective	Logistic regression	Inverse relationship
Ren et al. [11]	People in Shanghai, China	Retrospective	Cox proportional hazard regression	Inverse relationship
Bi et al. [20]	Experiment, mice model	Experimental data	Student's *t*-test	Positive correlation
Passarella et al. [22]	NA	Review	NA	Positive correlation

**Table 2:** Summary of microarray experiments used in this study.

Accession nos.	Platform	No. of diseased	No. of controls	Reference
*Alzheimer's disease cohort*				
GSE4757	GPL570	10 (entorhinal cortex)	10 (paired)	Dunckley et al. [30]
GSE48350	GPL570 (including 4 brain regions)	80	173	Berchtold et al. [31]
GSE5281	GPL570 (6 brain regions)	87	74	Liang et al. [32]

*Lung cancer cohort*				
GSE18842	GPL570	46	45 (paired)	Sanchez-Palencia et al. [33]
GSE102287	GPL570	32	34	Mitchell et al. [34]
GSE118370	GPL570	6	6	Xu et al. [35]
GSE19188	GLP570	94	62	Hou et al. [36]
GSE19804	GLP570	60	60	Lu et al. [37]
GSE103888	GLP570	13	6	Kuo et al. [38]

## Data Availability

Raw data of the Alzheimer's disease cohort used in the study were downloaded from the Gene Expression Omnibus (GEO: https://www.ncbi.nlm.nih.gov/geo/) repository under accession numbers GSE4757, GSE48350, and GSE5281. Microarray experiments for the lung cancer cohort were under accession numbers GSE18842, GSE102287, GSE19804, GSE19188, GSE103888, and GSE118370.
